# Advances in Early Detection of Melanoma and the Future of At-Home Testing

**DOI:** 10.3390/life13040974

**Published:** 2023-04-09

**Authors:** Zachary R. Garrison, Connor M. Hall, Rosalyn M. Fey, Terri Clister, Nabeela Khan, Rebecca Nichols, Rajan P. Kulkarni

**Affiliations:** 1 Department of Dermatology, Oregon Health & Science University, Portland, OR 97239, USA; 2 Cancer Early Detection Advanced Research Center (CEDAR), Portland, OR 97239, USA; 3 Knight Cancer Institute, Oregon Health and Science University, Portland, OR 97239, USA; 4 Operative Care Division, U.S. Department of Veterans Affairs Portland Health Care System, Portland, OR 97239, USA

**Keywords:** melanoma, early-detection, self-screening, primary-care, artificial intelligence

## Abstract

The past decade has seen numerous advancements in approaches to melanoma detection, each with the common goal to stem the growing incidence of melanoma and its mortality rate. These advancements, while well documented to increase early melanoma detection, have also garnered considerable criticism of their efficacy for improving survival rates. In this review, we discuss the current state of such early detection approaches that do not require direct dermatologist intervention. Our findings suggest that a number of at-home and non-specialist methods exist with high accuracy for detecting melanoma, albeit with a few notable concerns worth further investigation. Additionally, research continues to find new approaches using artificial intelligence which have promise for the future.

## 1. Introduction

In the United States, melanoma incidence has increased by 320% since 1975 [1]. Melanoma incidence has also increased globally [2]; this contrasts with overall cancer incidence, which has remained stable or partially declined [3]. As the fifth most common type of cancer in the US, there has been considerable effort to improve melanoma diagnostic capabilities, particularly at earlier stages. Direct access to dermatological screenings (as opposed to screenings by other types of physicians) correlates with earlier detection of melanoma and improved long-term survival [4]. In a large study on German patients diagnosed with melanoma, those who had participated in a dermatological screening program had higher survival rates than those who did not [5]. However, large-scale retrospective analysis has shown that these efforts have not been effective in stemming melanoma mortality rates [6].

While one approach for melanoma detection is via a dermatologist’s diagnosis, reliance on specialists can make it more difficult for those most in need of treatment to obtain care due to accessibility difficulties, thus leading to delays. Earlier identification of the disease (regardless of stage) has been shown to correlate with notably higher rates of survival [7]. These concerns have prompted the creation of several alternative approaches for early melanoma detection that do not require direct specialist involvement. Most efforts have gone into advocating for self-body screenings; however, some research points to difficulties in effectively teaching the criteria for identifying melanomas to patients [8,9]. These self-screening campaigns are particularly important for catching early signs of the disease [10]. In addition to survival benefits, the detection of melanoma at the early stages of disease progression is also associated with significant cost savings [11,12]. One study found that it is 1000-2000% more expensive to treat T1a and T4b tumors, respectively, compared to early-stage disease [13]. Despite critiques that the incurred costs of screening additional suspicious lesions will exceed any savings retained by not needing to treat later-stage disease, evidence has shown that early detection methods such as screening programs and dermatological screenings are cost-effective [14,15,16].

Intriguingly, campaigns to increase melanoma screenings by dermatologists found that such campaigns mostly resulted in increased screening rates for young women [17,18]. While this population has been observed to be at risk in youth demographics [19,20], it contrasts strongly with the elderly male population that is statistically the most at risk for melanoma [21,22]. Such a contradiction is a reason why further discussion of these melanoma detection methods is so important.

In this review, we discuss a variety of early detection methods for melanoma, including and beyond self-screening (Table 1). Given its limited discussion in the literature, we chose to analyze methods which are available for patients outside of dermatology clinics. Such approaches include the use of dynamic new technologies and deep learning artificial intelligence. Considering their importance in initial melanoma detection, we also discuss the roles that primary care physicians (PCPs) and general practitioners can play in directing melanoma cases.

## 2. Methods

Our goal for this review was to investigate which melanoma detection methods were available that did not require direct consultation with a dermatologist. To do so, articles were chosen for this literature review based on word relevancy to topics regarding at-home testing and primary care melanoma detection methods. The articles with direct relevance were used as a resource for finding other articles and expanding the discussion of certain methods. Certain papers were screened out in favor of more updated articles that had been published within the past 5–7 years. This focus on the literature was intended to make sure the review included the most up-to-date information regarding efficacy analysis and modern survey styles. Some articles included in this review were published prior to the aforementioned cutoff due to their direct relevancy to the addressed topics, and no better substitutes with more updated information were found.

## 3. Early Detection at Home

Developing new strategies that target unmet needs from previous campaigns is important for improving prognoses and outcomes for melanoma patients. Men tend have worse prognoses for melanoma [23], and are more likely to develop it during their lifetimes [22], but often delay seeking help for symptoms [24]. This phenomenon creates a barrier to early detection in healthcare settings, as those at the greatest risk are less likely to seek help for early symptoms. Considering this barrier and the importance of early detection, strategies to incorporate high-risk populations should ideally be accessible at home, before prospective patients seek primary care help.

Because of the visible nature of many melanomas, self-screening is already a major focus of several early-detection campaigns. Organizations such as the Melanoma Research Alliance [25], the AIM foundation [26], the Melanoma Research Foundation [27], and the American Melanoma Foundation [28] provide easily accessible resources that can guide the general public regarding how to conduct their own skin checks.

### 3.1. Naked Eye SSEs

Skin self-examinations (SSEs) are a widely-supported strategy requiring minimal equipment, and give the general public agency in their own cancer prevention. Studies have shown that such examinations can be effective in detecting earlier-stage melanoma reoccurrence [23]. These examinations track an individual’s moles using a simple, acronymic mnemonic device known as the “ABCDE Method” [29]. Each letter in this acronym stands for a key characteristic of a mole which may be noticeably altered with the presence of a cancerous lesion (Table 2).

The ABCDE method was initially developed at New York University in 1985 as an education tool aimed at helping physicians and the general public to differentiate between common moles and cancers [30]. Despite studies showing laypersons’ inability to produce accurate or consistent evaluations of skin lesions using the ABC criteria [31], this method can still be a useful tool for physicians [32,33].

A major strength of this SSE method is its ability to be performed anywhere an individual has adequate time and privacy. Additionally, there are data supporting this method’s efficacy in increasing melanoma detection when performed multiple times annually at home [34]. Another strength of the ABCDE method is the ease of modifying it to add additional letters that incorporate future elements worthy of consideration—a strategy that is already being implemented [35]. Individuals are also far more likely to recognize abnormalities in their own skin than a clinician is, as the clinician may only observe the patient once or twice in a year.

While the ABCDE method clearly defines important characteristics to observe during an SSE, there is no standardization for how examinations are conducted. As a result, there can be significant disparities in each examiner’s comprehensiveness. Less than eight percent of individuals performing SSEs check every body part detailed in the protocols, with the average self-examiner only checking two-thirds of their body [36]. It has also been observed that self-detected melanomas are more likely to be thicker and higher-risk cancers with poor prognoses [37]. Such data would suggest that self-screenings do not always increase survival outlook, as most individuals cannot positively identify the melanoma until it has already grown to a dangerous state. This somewhat contradicts the study mentioned previously [32], which showed that patients with a history of melanoma detected reoccurrences at earlier stages of disease. A possible explanation for this phenomenon is that patients with no prior experience with melanoma do not have the same sensitivity or ability to detect early-stage disease compared to patients with a history of melanoma.

Despite evidence of inconsistencies in laypersons’ ability to consistently use the ABC criteria to identify melanoma [31], this simple framework is a useful starting point for public outreach programs, but needs refinement to allow the average person to reliably identify suspicious lesions. Similar to the ABCDE method is the seven-point Glasgow checklist (7PCL), which has been recommended for use by The National Institute for Health and Care Excellence [38] and utilized by many physicians, particularly in the UK [39]. The 7CPL identifies seven key criteria for differentiating benign and cancerous moles, and suggests a referral to a specialist if a score of ≥3 is achieved [40] (Table 3).

As another SSE tool able to be used by the general public, the weighted 7PCL has shown to be more sensitive than the ABCDE method when employed by physicians [41]. The 7PCL has also been the focus of several awareness and self-screening campaigns in the past, most notable in the United Kingdom [42,43]. Despite the developmental intentions and their use in these campaigns, there is a need for studies measuring the efficacy of this method as a self-screening tool.

### 3.2. The Ugly Duckling Method

Another method available for individuals to use is to look for “ugly duckling signs”—moles which appear different from other moles on the same individual [44]. Generally benign nevi in an individual’s skin share common visual characteristics [45], and comparing all of a patient’s nevi has the potential to reduce the number of biopsies by a factor of seven as opposed to analyzing each nevi on an individual basis [46]. This is possibly the simplest diagnostic tool that can be given to individuals performing SSEs, and can be quickly communicated to patients through primary care physicians or awareness campaigns. However, this simplicity does not convey any additional characteristics of which to be conscious, and leaves additional room for certain populations to downplay cancer symptoms in order to delay seeking a professional opinion. Instead of replacing other methods, there is value in using ugly duckling signs to compliment other diagnostic tools. For example, the ABCDE method can be modified into the ABCDEF method, which includes an additional letter to represent “funny looking moles” [47].

Despite widespread encouragement of using naked-eye SSE to catch skin cancers in earlier stages, there is still a need for evidence to support its viability reducing patient mortality [48]. Additionally, intentional SSE protocols given to patients are not necessarily more effective than casual skin checks [23]. While SSEs can be useful for the rudimentary identification of a suspicious lesion, any attempt to distinguish between classes of diseases such as seborrheic keratoses and melanoma are beyond the ability of the average individual [33]. However, educational intervention-based approaches can be important tools to improve efficacy in these procedures [49]. SSEs, when used by non-dermatologists, may be equally sensitive in detecting in situ lesions vs. more developed forms [50], meaning that SSE strategies may need to adjust observation parameters to avoid missing skin cancer cases in their early stages. The downstream effect of increased identification of all suspicious lesions, both cancerous and benign, could overwhelm dermatology clinics with referrals.

Overall, naked-eye SSEs, as a tool to improve prognoses, have not produced reliable results. An element to consider is the role of skin awareness associated with an initial melanoma diagnosis, familial history of skin cancer, and individual interest in SSE procedures [51]. Skin awareness due to SSE is independently associated with improved outcome for patients [23], and, thus, should be a focus when standardizing SSE procedures and education in the future.

Although some believe that self-examinations are correlated more with over diagnosing than improved outcomes [52], others attest that overdiagnosis should not be the primary measure of early-detection efforts [53]. Additionally, in women at risk for melanoma, those who undergo online skin check training and have access to a dermatologist through a telehealth platform make fewer appointments for benign moles compared to those without these resources, suggesting that overdiagnosis may be attenuated by SSE education [54].

Variable at-home SSE performance and quality complicate our understanding of whether SSE is a useful tool for early detection. Among both melanoma survivors and those at risk for melanoma, only one-third to two-thirds of patients perform regular SSEs [36,55]. Further analysis of SSE among patients recovering from past melanoma shows a steady decline in the rate of SSE over time, despite the elevated risk among patients with a history of melanoma [51]. Similarly, an online survey of 88 members of the general population in Australia reported that two-thirds of the study’s participants regularly checked their skin [56]. Further confounding the evaluation of SSE as an early detection method is the difficulty of quantifying the quality of skin checks. Among those who do perform SSE, the measured comprehensiveness of the exam depends on the metrics used, such as number of body parts checked [33], or whether patients performed the SSE naked, with a mirror, with help from another person, and if palms/soles and new/existing nevi were examined [38].

Recent research efforts have shown that educational aids improve SSE in terms of both performance and quality. An SSE intervention strategy which included web-based education and a set of instructions/guide for SSE led to more thorough skin checks in at-risk participants [57]. Additionally, it has been shown that educational programs on skin self-examinations in schools may help to establish the use of sunscreen and continued self-examination behaviors which are helpful for early melanoma detection [58]. In a similar vein, game-based training on the ABCD (E excluded for training on static images) or UDS (ugly duckling sign) methods of SSE increased accurate melanoma identification in a study of the general population in the U.S. [59], suggesting that educational efforts based on established SSE aids may improve the detection of suspicious melanocytic lesions.

## 4. Primary Care Physician and General Practitioner Methods

While at-home testing is useful for the early detection of melanoma, it would be a mistake to leave out the role of primary care physicians (PCPs) and general practitioners in the discussion of early melanoma detection. Even when a patient may self-identify a suspicious mole, they will generally seek care first from their PCP, who may then refer to a specialist. Among all cancer types, melanoma has the shortest interval between initial clinical observation and referral [60], suggesting that most PCPs play an active role in recognizing signs of the disease. In doing so, these non-specialist physicians are charged with the challenging task of serving as a gatekeeper for further treatment without the full training necessary for melanoma identification. Hence, it is important to analyze the tools that are available to PCPs for identifying suspicious lesions, as well as the historical success rate of their referrals.

### 4.1. Dermatoscopes

While suspicious lesions can oftentimes be identified without the aid of any tools, one of the most effective ways to visually confirm the suspicious features associated with melanoma is by using a dermatoscope. The dermatoscope, invented in 1989 [59], operates as a specialized magnification device that allows physicians to observe skin areas with high detail, and can now be attached to cameras to take high-resolution photos [61]. The use of this device has shown to be extremely effective in the identification of melanoma amongst both dermatology specialists and general practitioners [62]. In a 2018 meta-analysis of a population of 42,000+ patients with lesions, the use of a dermatoscope increased physicians’ sensitivity to melanoma from 76% to 92% [63]. Similar findings were also observed in a cross-disciplinary analysis of both specialists and non-specialists [64]. These studies demonstrate the importance of integrating dermatoscope training into PCP education, and that efforts to promote dermatoscope use in their clinics may drastically improve the broader population’s ability to identify melanoma earlier. While some may contest that the additional cost associated with training and implementing dermatoscope use in PCP clinics may exceed any savings associated with earlier detection, a 2014 study observed cost savings in patient treatment which were in excess of any costs involved with PCPs using dermatoscopes [65]. While the technical knowledge may be challenging, research suggests that most PCPs would only need a single day of hands-on training to become proficient users of a dermatoscope [66].

### 4.2. Electronic Tools

Although the use of the dermatoscope has been shown to be quite useful in the hands of those without specialized dermatology training, there is still the consideration that these non-specialists may not have the expertise needed to optimally identify and detect suspicious lesions. To ameliorate their lack of expertise, various software tools have been developed to help PCPs to manage their patients’ skin health. Sequential digital dermoscopy imaging (SDDI) involves the collection and comparison of images of skin obtained via dermatoscope over an extended period of time to aid in the observation changes to the lesion’s physical traits. Initial analysis has shown that the employment of SDDI leads to cost savings in treatment which are far beyond the cost of using SDDI tools [67]. Additionally, incorporating SDDI alongside dermatoscope use resulted in >97% correct management of malignant pigmented lesions and melanoma by general practitioners in a 2009 British study [68].

SDDI is not the only available electronic tool that is designed to improve melanoma detection. A variety of image analysis software has been developed which provide more in-depth analyses of mole images. Spectrophotometric intracutaneous analysis (SIAscopy) is a non-invasive image analysis approach intended to diagnose pigmented skin lesions [69]. This tool has been designed not only for the use of dermatologists, but for non-specialized physicians as well. The use of SIAscopy has been shown to have an 85.7% sensitivity and 65.4% specificity to melanoma when used in a primary care setting [70].

An alternative approach to increasing a PCP’s ability to positively identify a suspicious lesion is via teledermatology. Teledermatology provides a streamlined approach through which general practitioners can seek expert opinions on their patients’ skin. By doing so, primary care physicians become able to speed up the rate at which patients can be consulted and adequate care can be directed. The use of teledermatology by PCPs in a retrospective study showed that over 50% of cases were able to be handled without the need of a dermatologist, which led to a staggering 78% reduction in the waiting time for in-person appointments [71]. Such an increase in specialist availability pays massive dividends, even beyond the observed patient cohort. An alternative type of teledermatology, known as “store-and-forward” teledermatology, has become increasingly popular in the past few decades. Rather than immediately sending dermatoscope images to a specialist, the store-and-forward method involves storing a series of images together in a database, such that a collective time-lapse of the skin region can be analyzed by a specialist [72]. This type of approach has been shown to be effective, and has led to an increased probability of successful cancer detection with fewer in-person visits [73,74]. While store-and-forward teledermatology has gained more traction, there is still room to improve its efficiency, as well as the public’s confidence in it. A longitudinal survey of clinics in Spain found that while teledermoscopy and teledermatology were continually becoming more popular, concerns over poor image quality, fear of error, and difficulty coordinating with PCPs were points of concern among both patients and their health providers [75]. Store-and-forward teledermatology is also not as commonly covered by insurance in the US, and is currently only available to those with Medicare in 27 states [76].

## 5. Debate over Efficacy of the New Method and Overdiagnosis

There is some contention as to whether many of these new approaches and initiatives actually improve melanoma detection and long-term survival. Some would argue that efforts to increase the detection of melanoma have not led to any survival outcomes or noticeable increases in melanoma. Incidence rates of less severe melanoma cases have increased (thinner melanomas and melanoma in situ) [77,78], despite melanoma-associated mortality remaining high [79,80]. Concerns have been raised regarding the efficacy of certain image analysis tools, such as the ones mentioned previously. MoleMate, a program built upon SIAscopy technology, has been observed to lead to a noticeably higher level of referrals, despite consistent disagreement between the program’s diagnosis and the results of expert assessment [81].

Despite these criticisms, there is a fair amount of evidence to show that non-specialists are more than capable of handling cases of suspicious lesions and making the correct referrals. An analysis which considered the differences in rural and urban melanoma cases found that rural areas with fewer specialists available had more lesions biopsied with no elevated harm or survival risk to their patients [82] compared to rural clinics at which specialists conducted biopsies less frequently. This suggests that some of the perceived risks that many may attribute to non-specialized physicians are without solid standing, and that increased involvement of PCPs in melanoma detection will result in an overall benefit to the patient population. Additional investigations of the variation in melanoma detection in dermatology versus non-specialist clinics have also supported the efficacy of general practitioners identifying melanoma. An interesting metric to consider in this comparison is the number needed to biopsy (NNB), which is defined as the number of biopsied lesions (as chosen by the directing physician) per positive melanoma diagnosis (validated by pathology). This ratio provides a measurement of the efficiency of identifying suspicious lesions. A 2020 study on melanoma referrals and detection in a variety of different specialties showed no statistically significant differences in the NNB achieved by dermatologists and non-dermatology specialists [83].

## 6. Future of Early Melanoma Screening

While we have discussed, at great length, the existing routes for melanoma detection, it is also important to consider where the field is heading and the emergent approaches. A common approach emerging in the literature is the use of artificial intelligence and deep learning models to diagnose melanoma using only the input of a dermatoscope image. Plenty of evidence has been presented that demonstrates that these programs are efficient in accurately diagnosing melanoma at similar levels to dermatologists [84,85,86,87,88]. Such imaging is collected by a primary care physician or other provider, then uploaded to software which analyzes a variety of features against a database of images of healthy and diseased skin [89]. A computer-aided diagnosis (CAD) application, developed for smartphone use, was validated in PCP clinics, demonstrating >80% accuracy and close to 90% sensitivity [90]. Another study examining 20 PCPs and 20 nurse practitioners found that AI assistance was significantly associated with higher diagnosis agreement with a dermatologist panel [91]. Dildar et al. provided a thorough discussion of the technical side of AI-based skin cancer detection and how the software is designed [92].

Such technologies are still very much in their infancy, and there is much room for improvement. There are several weaknesses associated with the existing AI technologies. One of these is the black-box nature of this type of software. Without an understanding of how the images are being analyzed and which features take the most precedence in the diagnosis, one could imagine a scenario in which a provider is debating between two diagnoses as a result of one key defining feature of a lesion, and turns to AI in hopes of clarification. However, the software may produce a completely different diagnosis without a justification as to why. Such an output would only confuse the provider further, and would not help to direct the patient towards treatment any sooner. AI provides an opportunity for non-specialists to gain dermatology-level insight into challenging diagnoses. Unfortunately, this requires the AI software to provide explanations regarding how it works, which most types do not. There is also the consideration of the ever-evolving metrics for melanoma detection. Similarly to other cancers, melanoma research continues to prompt changes in the protocols we use for diagnosis. This suggests that any programs designed to diagnose melanoma must be consistently updated to accommodate the existing research. Given this logic, most would be surprised to find that many of the most popular melanoma diagnostic apps have not been updated in several years [93].

Such apps also have cost barriers in place. Some smartphone apps designed for patient use have a monthly subscription fee and require the purchase of a dermatoscope attachment, which ranges from USD 99–299 [94]. Such large upfront costs restrict the availability of these technologies from those in lower socioeconomic groups who may not have access to a smartphone, let alone a USD 100+ app. Additionally, those most likely to pay for such tools are far more likely to be those with a history of disease/high risk of reoccurrence as opposed to the general public, further limiting their widespread utility. The War on Melanoma program at Oregon Health and Science University does address this issue with its Sklip^®^ at-home dermoscopy program, which loans dermatoscope attachments to patients [95]. In addition, such efforts could be incorporated into community outreach efforts to increase access.

The origin of the images used for developing this technology is also a point of concern. A comprehensive analysis of several deep learning algorithms showed that all of the images used to create said algorithms were obtained from only three of the fifty states [96]. Those three states (California, Massachusetts, and New York) are composed of largely white population at the higher end of the socioeconomic ladder. The fact that such a skewed subgroup was used for the creation of these deep learning technologies likely indicates some ingrained biases that would limit their applicability to non-white populations. Such an issue is part of a larger conversation regarding bias in AI that points to pivotal weakness in its widespread efficacy in at-risk populations [97].

## 7. Conclusions

The past few decades have seen significant advancements in efforts and technologies to enable earlier detection of melanoma and to increase survival outcomes. These efforts have largely been focused towards campaigns for at-home detection via patient self-screenings, increased training and resources for melanoma detection by non-specialists, and new technological methods for melanoma diagnosis. These new approaches to melanoma detection all serve the collective goal of reducing the need for dermatology-driven diagnosis, thereby opening up clinical resources for those most in need of treatment. While the field has made significant progress in this pursuit, the outlined methods for melanoma detection have accuracy concerns stemming from lack of expertise and internal biases. Such drawbacks have fostered criticism, including the notion that efforts to increase melanoma detection have only led to overdiagnosis [98] and increased costs, without the desired survival outcomes [99,100,101,102].

Such critiques fail to recognize that we do not yet understand how to stratify these melanoma cases, as more research into the underlying biology is necessary. Until then, abandoning the pursuit of additional melanoma screening will not reduce the overall morbidity of the disease, but may lead to later diagnoses. The research outlined above clearly demonstrates that these additional methods for melanoma detection are effective in identifying melanoma earlier, albeit in cases of lower-risk disease. This suggests that mobilization of the non-specialist population is possible, but requires tweaking to actually increase the survival outcomes in the melanoma population. Equal access across the socioeconomic spectrum must also be considered, as many of the previously outlined methods involve costly methods. Those without the resources to regularly meet with a physician of any kind or to use a smartphone would find many of the options discussed in this review out of their reach. Rural medicine and low-cost approaches must be a consideration in the next phase of melanoma detection design.

Additional studies are required to determine whether these early detection campaigns confer any survival benefits to these patients relative to those who would otherwise wait until more severe clinical symptoms arose. Such findings would be extremely helpful in settling the overdiagnosis debate currently raging in the field of dermatology. The field should also be investing their efforts towards better training and educational resources for the general public, as well as PCPs, to more effectively identify melanoma. Such training must also be more efficiently targeted, such that it reaches the most at-risk populations with reduced access to dermatologists. The ability of PCPs to step into more independent melanoma-diagnosing roles has the potential to dramatically increase early detection of melanoma and help to alleviate the workloads of many overscheduled dermatologists.

In addition to in-person melanoma detection methods, AI also presents an intriguing opportunity for melanoma detection without reliance on specialists. The high efficacy rates are promising, although they come with the major caveat that is the mysterious nature of the origin and function of the software. Greater transparency in these deep-learning technologies is required for them to become more socially acceptable and more useful for non-specialist use. Efforts must also be made to address the imbued racial and socioeconomic biases in the software such that they can serve to improve, rather than reinforce, the healthcare disparities already present in the diagnosis of melanoma [103,104,105].

## Figures and Tables

**Table 1:** Melanoma detection summary.

Method	Target Audience	Pros	Cons
**Skin Self-Examinations (ABCDE/Seven-Point Glasgow/Ugly Duckling)**	General public	- No cost - Quick - No specialized equipment or training - Can be conducted regularly	- Inconsistent results regarding efficacy - General public not capable of distinguishing nuances in skin conditions - May contribute to needless referrals and over-diagnosing
**Dermatoscope Inspection**	Primary care physicians	- Effective tool with proven advantage over naked-eye examination - Minimal training required for it to be effective in the hands of non-specialists	- Cost associated with purchase - Requires training to use
**Smartphone Apps**	General public and primary care physicians	- Easy to use - Accessible to anyone with a smartphone	- Can be expensive - Software not regularly updated
**Artificial Intelligence-Based Image Analysis**	General public and primary care physicians	- Utilizes evolving and improving technology to identify suspicious lesions - Can be easily updated as new metrics are discovered - Requires minimal input from the user	- Black box nature of AI software - Racial bias of software designed using non-diversified images
**Sequential Digital Dermoscopy**	General Public and primary care physicians	- Simple way to track suspicious lesions for individuals without technical experience - Allows for outreach to specialists if needed in the future	
**Teledermatology**	General public	- “Store and forward” approach helps individuals to receive quick consultations from specialists - All the benefits of receiving a dermatologist’s analysis without visiting the clinic - Increased likelihood of earlier cancer detection	- Requires input from dermatologists; can overwhelm specialists if broadly applied - Not covered by insurance in many states

**Table 2:** The ABCDE Method.

Letter	Characteristic
**A**	**Asymmetry**—benign moles typically have symmetric or uniform shapes, while cancerous moles are often asymmetric or irregular.
**B**	**Border**—benign moles have round and distinct borders, whereas cancerous moles often have asymmetric or jagged borders.
**C**	**Color**—benign moles tend to be a single color, while cancerous moles are often composed of multiple shades or colors at different parts of the mole.
**D**	**Diameter**—cancerous moles are typically over six millimeters in diameter. This is approximately the diameter of a common pencil.
**E**	**Evolving**—Unlike benign moles, cancerous moles often change in size, shape, and color over time.

**Table 3:** The seven-point Glasgow checklist.

Characteristic	Weighted Score Value
Change in the size of a lesion	2 points
Irregularity in the shape of a lesion	2 points
Irregularity in the color of a lesion	2 points
Inflammation in or around the lesion	1 point
Alteration in sensation of the lesion	1 point
The lesion is large in size, or has a diameter larger than seven millimeters	1 point
Oozing or crusting on or around the lesion	1 point

## Data Availability

Not applicable as no new data were created for this article.

## References

[1] Saginala K., Barsouk A., Aluru J.S., Rawla P., Barsouk A. (2021). Epidemiology of Melanoma. Med. Sci..

[2] Carr S., Smith C., Wernberg J. (2020). Epidemiology and risk factors of melanoma. Surg. Clin. N. Am..

[3] Tripp M.K., Watson M., Balk S.J., Swetter S.M., Gershenwald J.E. (2016). State of the science on prevention and screening to reduce melanoma incidence and mortality: The time is now. CA Cancer J. Clin..

[4] Pennie M.L., Soon S.L., Risser J.B., Veledar E., Culler S., Chen S.C. (2007). Melanoma outcomes for Medicare patients: Association of stage and survival with detection by a dermatologist vs a nondermatologist. Arch. Derm..

[5] Datzmann T., Schoffer O., Meier F., Seidler A., Schmitt J. (2022). Are patients benefiting from participation in the German skin cancer screening programme? A large cohort study based on administrative data. Br. J. Derm..

[6] Stang A., Jöckel K.H. (2016). Does skin cancer screening save lives? A detailed analysis of mortality time trends in Schleswig-Holstein and Germany. Cancer.

[7] Leiter U., Buettner P.G., Eigentler T.K., Forschner A., Meier F., Garbe C. (2010). Is detection of melanoma metastasis during surveillance in an early phase of development associated with a survival benefit?. Melanoma Res..

[8] Borland R., Marks R., Noy S. (1992). Public knowledge about characteristics of moles and melanomas. Aust. J. Public Health.

[9] Lawson D.D., Moore D.H., Schneider J.S., Sagebiel R.W. (1994). Nevus counting as a risk factor for melanoma: Comparison of self-count with count by physician. J. Am. Acad. Dermatol..

[10] Hamidi R., Peng D., Cockburn M. (2010). Efficacy of skin self-examination for the early detection of melanoma. Int. J. Dermatol..

[11] Elliott T.M., Whiteman D.C., Olsen C.M., Gordon L.G. (2017). Estimated Healthcare Costs of Melanoma in Australia over 3 Years Post-Diagnosis. Appl. Health Econ. Health Policy.

[12] Serra-Arbeloa P., Rabines-Juárez Á., Álvarez-Ruiz M., Guillén-Grima F. (2017). Cost of Cutaneous Melanoma by Tumor Stage: A Descriptive Analysis. Estudio descriptivo de costes en melanoma cutáneo de diferentes estadios. Actas Dermosifiliogr..

[13] Alexandrescu D. (2009). Melanoma costs: A dynamic model comparing estimated overall costs of various clinical stages. Derm. Online J..

[14] Girgis A., Clarke P., Burton R.C., Sanson—Fisher R.W. (1996). Screening for melanoma by primary health care physicians: A cost-effectiveness analysis. J. Med. Screen..

[15] Freedberg K.A., Geller A.C., Miller D.R., Lew R.A., Koh H.K. (1999). Screening for malignant melanoma: A cost-effectiveness analysis. J. Am. Acad. Dermatol..

[16] Losina E., Walensky R.P., Geller A., Beddingfield F.C., Wolf L.L., Gilchrest B.A., Freedberg K.A. (2007). Visual screening for malignant melanoma: A cost-effectiveness analysis. Arch. Derm..

[17] van der Leest R., de Vries E., Bulliard J.-L., Paoli J., Peris K., Stratigos A., Trakatelli M., Maselis T., Šitum M., Pallouras A. (2011). The Euromelanoma skin cancer prevention campaign in Europe: Characteristics and results of 2009 and 2010. J. Eur. Acad. Derm. Venereol..

[18] Geller A.C., Zhang Z., Sober A.J., Halpern A.C., Weinstock M.A., Daniels S., Miller D.R., Demierre M.-F., Brooks D., Gilchrest B.A. (2003). The first 15 years of the American Academy of Dermatology skin cancer screening programs: 1985–1999. J. Am. Acad. Derm..

[19] Paulson K.G., Gupta D., Kim T.S., Veatch J.R., Byrd D.R., Bhatia S., Wojcik K., Chapuis A.G., Thompson J.A., Madeleine M.M. (2020). Age-Specific Incidence of Melanoma in the United States. JAMA Dermatol..

[20] Liu-Smith F., Ziogas A. (2020). Age-dependent interaction between sex and geographic ultraviolet index in melanoma risk. J. Am. Acad. Dermatol..

[21] Cancer Research UK Melanoma Skin Cancer Incidence Statistics. https://www.cancerresearchuk.org/health-professional/cancer-statistics/statistics-by-cancer-type/melanoma-skin-cancer/incidence#heading-Zero.

[22] Bellenghi M., Puglisi R., Pontecorvi G., De Feo A., Carè A., Mattia G. (2020). Sex and Gender Disparities in Melanoma. Cancers.

[23] Paddock L.E., Lu S.E., Bandera E.V., Rhoads G.G., Fine J., Paine S., Barnhill R., Berwick M. (2016). Skin self-examination and long-term melanoma survival. Melanoma Res..

[24] David J. (2021). Men’s Health and the Primary Care Physician.

[25] Melanoma Research Alliance Early Warning Signs of Melanoma and Other Skin Cancers. https://www.curemelanoma.org/about-melanoma/educate-yourself/know-what-to-look-for.

[26] AIM at Melanoma Foundation How to Do a Skin Self-Examination. https://www.aimatmelanoma.org/melanoma-101/early-detection-of-melanoma/how-to-do-a-skin-self-examination.

[27] Melanoma Research Foundation What Melanoma Looks Like. https://melanoma.org/melanoma-education/what-melanoma-looks-like.

[28] The American Melanoma Foundation. https://melanomafoundation.org.

[29] Rigel D.S., Friedman R.J., Kopf A.W., Polsky D. (2005). ABCDE—An Evolving Concept in the Early Detection of Melanoma. Arch. Dermatol..

[30] Rigel D., Russak J., Friedman R. (2010). The Evolution of Melanoma Diagnosis: 25 Years Beyond the ABCDs. CA Cancer J. Clin..

[31] Aldridge R., Zanotto M., Ballerini L., Fisher R., Rees J. (2011). Novice Identification of Melanoma: Not Quite as Straightforward as the ABCDs. Acta Derm. Venereol..

[32] Healsmith M., Bourke J., Osborne J., Graham-Brown R. (1994). An evaluation of the revised seven-point checklist for the early diagnosis of cutaneous malignant melanoma. Br. J. Dermatol..

[33] Thomas L., Tranchand P., Berard F., Secchi T., Colin C., Moulin G. (1998). Semiological Value of ABCDE Criteria in the Diagnosis of Cutaneous Pigmented Tumors. Dermatology.

[34] Titus L., Clough-Gorr K., Mackenzie T., Perry A., Spencer S., Weiss J., Abrahams-Gessel S., Ernstoff M. (2013). Recent skin self-examination and doctor visits in relation to melanoma risk and tumour depth. Br. J. Dermatol..

[35] Melanoma: Clinical Features and Diagnosis. https://www.medilib.ir/uptodate/show/15806.

[36] Manne S.L., Heckman C.J., Kashy D., Lozada C., Gallo J., Ritterband L., Coups E.J. (2020). Prevalence and correlates of skin self-examination practices among cutaneous malignant melanoma survivors. Prev. Med. Rep..

[37] De Giorgi V., Grazzini M., Rossari S., Gori A., Papi F., Scarfi F., Savarese I., Gandini S. (2012). Is skin self-examination for cutaneous melanoma detection still adequate? A retrospective study. Dermatology.

[38] Jones O.T., Ranmuthu C.K.I., Hall P.N., Funston G., Walter F.M. (2020). Recognising Skin Cancer in Primary Care. Adv. Ther..

[39] Pannebakker M.M., Mills K., Johnson M., Emery J.D., Walter F.M. (2019). Understanding implementation and usefulness of electronic clinical decision support (eCDS) for melanoma in English primary care: A qualitative investigation. BJGP Open.

[40] Walter F.M., Prevost A.T., Vasconcelos J., Hall P.N., Burrows N.P., Morris H.C., Kinmonth A.L., Emery J.D. (2013). Using the 7-point checklist as a diagnostic aid for pigmented skin lesions in general practice: A diagnostic validation study. Br. J. Gen. Pract..

[41] Mittal A., Pushpam D., Bakhshi S. (2020). Management of advanced melanoma in the current era: A medical oncology perspective for the Indian scenario. Natl. Med. J. India.

[42] Graham-Brown R., Osborne J.E., London S.P., Fletcher A., Shaw D., Williams B., Bowry V. (1990). The initial effects on workload and outcome of a public education campaign on early diagnosis and treatment of malignant melanoma in Leicestershire. Br. J. Dermatol..

[43] Doherty V., MacKie R. (1988). Experience of a public education programme on early detection of cutaneous malignant melanoma. Br. Med. J..

[44] Cantisani C., Ambrosio L., Cucchi C., Meznerics F.A., Kiss N., Bánvölgyi A., Rega F., Grignaffini F., Barbuto F., Frezza F. (2022). Melanoma Detection by Non-Specialists: An Untapped Potential for Triage?. Diagnostics.

[45] Zhang Y., Ali K., George J.A., Reichenberg J.S., Fox M.C., Adamson A.S., Tunnell J.W., Markey M.K. (2021). Toward automated assessment of mole similarity on dermoscopic images. J. Med. Imaging.

[46] Gaudy-Marqueste C., Wazaefi Y., Bruneu Y., Triller R., Thomas L., Pellacani G., Malvehy J., Avril M.-F., Monestier S., Richard M.-A. (2017). Ugly Duckling Sign as a Major Factor of Efficiency in Melanoma Detection. JAMA Dermatol..

[47] Jensen J.D., Elewski B.E. (2015). The ABCDEF Rule: Combining the “ABCDE Rule” and the “Ugly Duckling Sign” in an Effort to Improve Patient Self-Screening Examinations. J. Clin. Aesthetic Dermatol..

[48] Ersser S., Effah A., Dyson J., Kellar I., Thomas S., McNichol E., Caperon E., Hewitt C., Muinonen-Martin A. (2019). Effectiveness of interventions to support the early detection of skin cancer through skin self-examination: A systematic review and meta-analysis. Br. J. Dermatol..

[49] Czajkowska Z., Hall N., Sewitch M., Wang B., Körner A. (2017). The role of patient education and physician support in self-efficacy for skin self-examination among patients with melanoma. Patient Educ. Couns..

[50] Duarte A.F., Sousa-Pinto B., Azevedo L.F., Barros A.M., Puig S., Malvehy J., Haneke E., Correia O. (2021). Clinical ABCDE rule for early melanoma detection. Eur. J. Dermatol..

[51] Coroiu A., Moran C., Bergeron C., Drapeau M., Wang B., Kezouh A., Ernst J., Batist G., Körner A. (2020). Short and long-term barriers and facilitators of skin self-examination among individuals diagnosed with melanoma. BMC Cancer.

[52] De Giorgi V., Papi F., Giorgi L., Savarese I., Verdelli A., Scarfì F., Gandini S. (2013). Skin self-examination and the ABCDE rule in the early diagnosis of melanoma: Is the game over?. Br. J. Dermatol..

[53] Kulkarni R., Wesley Y., Leachman S. (2022). To Improve Melanoma Outcomes, Focus on Risk Stratification, Not Overdiagnosis. JAMA Dermatol..

[54] Robinson J.K., Wahood S., Ly S., Kirk J., Yoon J., Sterritt J., Gray E., Kwasny M. (2021). Melanoma detection by skin self-examination targeting at-risk women: A randomized controlled trial with telemedicine support for concerning moles. Prev. Med. Rep..

[55] Sarikaya Solak S., Yondem H., Cicin I. (2020). Evaluating sun protection behaviors and skin self-examination practices among the family members of melanoma patients in Turkey: A cross-sectional survey study. Dermatol. Ther..

[56] Koh U., Horsham C., Soyer H.P., Loescher L.J., Gillespie N., Vagenas D., Janda M. (2019). Consumer Acceptance and Expectations of a Mobile Health Application to Photograph Skin Lesions for Early Detection of Melanoma. Dermatology.

[57] Manne S.L., Marchetti M.A., Kashy D.A., Heckman C.J., Ritterband L.M., Thorndike F.P., Viola A., Lozada C., Coups E.J. (2022). mySmartCheck, a Digital Intervention to Promote Skin Self-examination Among Individuals Diagnosed with or at Risk for Melanoma: A Randomized Clinical Trial. Ann. Behav. Med..

[58] Hubbard G., Kyle R.G., Neal R.D., Marmara V., Wang Z., Dombrowski S.U. (2018). Promoting sunscreen use and skin self-examination to improve early detection and prevent skin cancer: Quasi-experimental trial of an adolescent psycho-educational intervention. BMC Public Health.

[59] Carcioppolo N., Kim S., Sanchez M., Mao B., Malova E., Ryan A., Lun D., Ewing C., Hu S. (2022). Evaluating a game-based randomized experiment to increase melanoma identification among adults living in the US. Soc. Sci. Med..

[60] Swann R., McPhail S., Witt J., Shand B., Abel G., Hiom S., Rashbass J., Lyratzopoulos G., Rubin G., The National Cancer Diagnosis Audit Steering Group (2018). Diagnosing cancer in primary care: Results from the national cancer diagnosis audit. Br. J. Gen. Pract..

[61] Katz B., Rabinovitz H. (2001). Introduction to Dermoscopy. Dermatol. Clin..

[62] Jones O.T., Jurascheck L.C., van Melle M., Hickman S., Burrows N.P., Hall P.N., Emery J., Walter F. (2019). Dermoscopy for melanoma detection and triage in primary care: A systematic review. BMJ Open.

[63] Dinnes J., Deeks J.J., Chuchu N., di Ruffano L.F., Matin R.N., Thomson D.R., Wong K.Y., Aldridge R.B., Abbott R., Fawzy M. (2018). Dermoscopy, with and without visual inspection, for diagnosing melanoma in adults. Cochrane Database Syst. Rev..

[64] Vestergaard M., Macaskill P., Holt P., Menzies S. (2008). Dermoscopy compared with naked eye examination for the diagnosis of primary melanoma: A meta-analysis of studies performed in a clinical setting. Br. J. Dermatol..

[65] Koelink C., Vermeulen K., Kollen B., de Bock G., Dekker J., Jonkman M., van der Heide W. (2014). Diagnostic accuracy and cost-effectiveness of dermoscopy in primary care: A cluster randomized clinical trial. J. Eur. Acad. Dermatol. Venereol..

[66] Jones O., Jurascheck L., Utukuri M., Pannebakker M., Emery J., Walter F. (2019). Dermoscopy, use in UK Primary care: A survey of GP’s with a special interest in dermatology. J. Eur. Acad. Dermatol. Venerol..

[67] Tromme I., Devleesschauwer B., Beutels P., Richez P., Praet N., Sacré L., Marot L., Van Eeckhout P., Theate I., Baurain J.-F. (2014). Selective use of sequential digital dermoscopy imaging allows a cost reduction in the melanoma detection process: A belgian study of patients with a single or a small number of atypical nevi. PLoS ONE.

[68] Menzies S., Emery J., Staples M., Davies S., McAvoy B., Fletcher J., Shahid K., Reid G., Avramidis M., Ward A. (2009). Impact of dermoscopy and short-term sequential digital dermoscopy imaging for the management of pigmented lesions in primary care: A sequential intervention trial. Br. J. Dermatol..

[69] Emery J.D., Hunter J., Hall P.N., Watson A.J., Moncrieff M., Walter F.M. (2010). Accuracy of SIAscopy for pigmented skin lesions encountered in primary care: Development and validation of a new diagnostic algorithm. BMC Dermatol..

[70] Sgouros D., Lallas A., Julian Y., Rigopoulos D., Zalaudek I., Longo C., Moscarella E., Simonetti V., Argenziano G. (2014). Assessment of SIAscopy in the triage of suspicious skin tumours. Ski. Res. Technol..

[71] Giavina-Bianchi M., Santos A.P., Cordioli E. (2020). Teledermatology reduces dermatology referrals and improves access to specialists. EClinicalMedicine.

[72] Marwaha S.S., Fevrier H., Alexeeff S., Crowley E., Haiman M., Pham N., Tuerk M.J., Wukda D., Hartmann M., Herrinton L.J. (2019). Comparative effectiveness study of face-to-face and teledermatology workflows for diagnosing skin cancer. J. Am. Acad. Dermatol..

[73] Mohan G., Molina G., Stavert R. (2018). Store and forward teledermatology improves dermatology knowledge among referring primary care providers: A survey-based cohort study. J. Am. Acad. Dermatol..

[74] Romero G., De Argila D., Ferrandiz L., Sánchez M., Vañó S., Taberner R., Pasquali P., De La Torre C., Alfageme F., Malvehy J. (2018). Practice Models in Teledermatology in Spain: Longitudinal Study, 2009–2014. Modelos de práctica de la teledermatología en España. Estudio longitudinal 2009–2014. Actas Dermo-Sifiliográficas.

[75] CCHP Telehealth Policy Trend Maps. https://www.cchpca.org/policy-trends.

[76] Glazer A.M., Winkelmann R.R., Farberg A.S., Rigel D.S. (2017). Analysis of Trends in US Melanoma Incidence and Mortality. JAMA Dermatol..

[77] Matsumoto M., Wack S., Weinstock M.A., Geller A., Wang H., Solano F.X., Kirkwood J.M., Ferris L.K. (2022). Five-Year Outcomes of a Melanoma Screening Initiative in a Large Health Care System. JAMA Dermatol..

[78] Welch H., Mazer B., Adamson A. (2021). The rapid rise in cutaneous melanoma diagnoses. N. Engl. J. Med..

[79] Herbert A., Koo M.M., Barclay M.E., Greenberg D.C., Abel G.A., Levell N.J., Lyratzopoulos G. (2020). Stage-specific incidence trends of melanoma in an English region, 1996–2015: Longitudinal analyses of population-based data. Melanoma Res..

[80] Watts C.G., McLoughlin K., Goumas C., van Kemenade C.H., Aitken J.F., Soyer H.P., Peñas P.F., Guitera P., Scolyer R.A., Morton R.L. (2021). Association Between Melanoma Detected During Routine Skin Checks and Mortality. JAMA Dermatol..

[81] Walter F.M., Morris H.C., Humphrys E., Hall P.N., Prevost A.T., Burrows N., Bradshaw L., Wilson E.C., Norris P., Walls J. (2012). Effect of adding a diagnostic aid to best practice to manage suspicious pigmented suspicious pigmented lesions in primary care: Randomised controlled trial. Br. Med. J..

[82] Murchie P., Adam R., Khor W.L., Raja E.A., Iversen L., Brewster D., Lee A.J. (2018). Impact of rurality on processes and outcomes in melanoma care: Results from a whole-Scotland melanoma cohort in primary and secondary care. Br. J. Gen. Pract..

[83] Privalle A., Havighurst T., Kim K., Bennett D.D., Xu Y.G. (2020). Number of skin biopsies needed per malignancy: Comparing the use of skin biopsies among dermatologists and nondermatologist clinicians. J. Am. Acad. Dermatol..

[84] Haenssle H.A., Fink C., Schneiderbauer R., Toberer F., Buhl T., Blum A., Kalloo A., Hassen A.B.H., Thomas L., Enk A. (2018). Man against machine: Diagnostic performance of a deep learning convolutional neural network for dermoscopic melanoma recognition in comparison to 58 dermatologists. Ann. Oncol..

[85] Phillips M., Marsden H., Jaffe W., Matin R.N., Wali G.N., Greenhalgh J., McGrath E., James R., Ladoyanni E., Bewley A. (2019). Assessment of Accuracy of an Artificial Intelligence Algorithm to Detect Melanoma in Images of Skin Lesions. JAMA Netw. Open.

[86] Brinker T.J., Hekler A., Enk A.H., Berking C., Haferkamp S., Hauschild A., Weichenthal M., Klode J., Schadendorf D., Holland-Letz T. (2019). Deep neural networks are superior to dermatologists in melanoma image classification. Eur. J. Cancer.

[87] Dick V., Sinz C., Mittlböck M., Kittler H., Tschandl P. (2019). Accuracy of Computer-Aided Diagnosis of Melanoma: A Meta-analysis. JAMA Dermatol..

[88] Phillips M., Greenhalgh J., Marsden H., Palamaras I. (2020). Detection of malignant melanoma using artificial intelligence: An observational study of diagnostic accuracy. Dermatol. Pract. Concept.

[89] Dascalu A., David E.O. (2019). Skin cancer detection by deep learning and sound analysis algorithms: A prospective clinical study of an elementary dermoscope. Discov. Sci..

[90] Giavina-Bianchi M., de Sousa R.M., Paciello V.Z.d.A., Vitor W.G., Okita A.L., Prôa R., Severino G.L.d.S., Schinaid A.A., Santo R.E., Machado B.S. (2021). Implementation of artificial intelligence algorithms for melanoma screening in a primary care setting. PLoS ONE.

[91] Jain A., Way D., Gupta V., Gao Y., Marinho G.D.O., Hartford J., Sayres R., Kanada K., Eng C., Nagpal K. (2021). Development and Assessment of an Artificial Intelligence–Based Tool for Skin Condition Diagnosis by Primary Care Physicians and Nurse Practitioners in Teledermatology Practices. JAMA Netw. Open.

[92] Dildar M., Akram S., Irfan M., Khan H.U., Ramzan M., Mahmood A.R., Alsaiari S.A., Saeed A.H.M., Alraddadi M.O., Mahnashi M.H. (2021). Skin Cancer Detection: A Review Using Deep Learning Techniques. Int. J. Environ. Res. Public Health.

[93] Kassianos A., Emery J., Murchie P., Walter F. (2015). Smartphone applications for melanoma detection by community, patient and generalist clinician users: A review. Br. J. Dermatol..

[94] Kong F.W., Horsham C., Ngoo A., Soyer H.P., Janda M. (2020). Review of smartphone mobile applications for skin cancer detection: What are the changes in availability, functionality, and costs to users over time. Int. J. Dermatol..

[95] OHSU War on Melanoma. https://www.ohsu.edu/war-on-melanoma/sklipr-home-dermoscopy.

[96] Kaushal A., Altman R., Langlotz C. (2020). Geographic Distribution of US Cohorts Used to Train Deep Learning Algorithms. JAMA.

[97] Parikh R.B., Teeple S., Navathe A.S. (2019). Addressing Bias in Artificial Intelligence in Health Care. JAMA.

[98] Dunn B.K., Woloshin S., Xie H., Kramer B.S. (2022). Cancer overdiagnosis: A challenge in the era of screening. J. Natl. Cancer Cent..

[99] Whiteman D.C., Olsen C.M., MacGregor S., Law M.H., Thompson B., Dusingize J.C., Green A.C., Neale R.E., Pandeya N. (2022). The effect of screening on melanoma incidence and biopsy rates. Br. J. Dermatol..

[100] Muzumdar S., Lin G., Kerr P., Grant-Kels J.M. (2021). Evidence concerning the accusation that melanoma is overdiagnosed. J. Am. Acad. Dermatol..

[101] Kurtansky N.R., Dusza S.W., Halpern A.C., Hartman R.I., Geller A.C., Marghoob A.A., Rotemberg V.M., Marchetti M.A. (2022). An Epidemiologic Analysis of Melanoma Overdiagnosis in the United States 1975–2017. J. Investig. Dermatol..

[102] Rubin R. (2020). Melanoma Diagnoses Rise While Mortality Stays Fairly Flat, Raising Concerns About Overdiagnosis. JAMA.

[103] Brunsgaard E., Jensen J., Grossman D. (2022). Melanoma in Skin of Color: Part II. Racial disparities, role of UV, and interventions for earlier detection. J. Am. Acad. Dermatol..

[104] Tripathi R., Archibald L.K., Mazmudar R.S., Conic R.R., Rothermel L.D., Scott J.F., Bordeaux J.S. (2020). Racial differences in time to treatment for melanoma. J. Am. Acad. Dermatol..

[105] Cortez J., Vasquez J., Wei M. (2021). The impact of demographics, socioeconomics, and health care access on melanoma outcomes. J. Am. Acad. Dermatol..

